# Investigating the Potential for Clinical Decision Support in Sub-Saharan Africa With AFYA (Artificial Intelligence-Based Assessment of Health Symptoms in Tanzania): Protocol for a Prospective, Observational Pilot Study

**DOI:** 10.2196/34298

**Published:** 2022-06-07

**Authors:** Marcel Schmude, Nahya Salim, Hila Azadzoy, Mustafa Bane, Elizabeth Millen, Lisa O’Donnell, Philipp Bode, Ewelina Türk, Ria Vaidya, Stephen Gilbert

**Affiliations:** 1 Ada Health GmbH Berlin Germany; 2 Muhimbili University of Health and Allied Sciences Dar es Salaam United Republic of Tanzania; 3 Else Kröner Fresenius Center for Digital Health, University Hospital Carl Gustav Carus Dresden, Technische Universität Dresden Dresden Germany

**Keywords:** differential diagnosis, artificial intelligence, clinical decision support systems, decision support, diagnostic decision support systems, diagnosis, Africa, low income, middle income, user centred design, user centered design, symptom assessment, chatbot, health app, prototype

## Abstract

**Background:**

Low- and middle-income countries face difficulties in providing adequate health care. One of the reasons is a shortage of qualified health workers. Diagnostic decision support systems are designed to aid clinicians in their work and have the potential to mitigate pressure on health care systems.

**Objective:**

The Artificial Intelligence–Based Assessment of Health Symptoms in Tanzania (AFYA) study will evaluate the potential of an English-language artificial intelligence–based prototype diagnostic decision support system for mid-level health care practitioners in a low- or middle-income setting.

**Methods:**

This is an observational, prospective clinical study conducted in a busy Tanzanian district hospital. In addition to usual care visits, study participants will consult a mid-level health care practitioner, who will use a prototype diagnostic decision support system, and a study physician. The accuracy and comprehensiveness of the differential diagnosis provided by the diagnostic decision support system will be evaluated against a gold-standard differential diagnosis provided by an expert panel.

**Results:**

Patient recruitment started in October 2021. Participants were recruited directly in the waiting room of the outpatient clinic at the hospital. Data collection will conclude in May 2022. Data analysis is planned to be finished by the end of June 2022. The results will be published in a peer-reviewed journal.

**Conclusions:**

Most diagnostic decision support systems have been developed and evaluated in high-income countries, but there is great potential for these systems to improve the delivery of health care in low- and middle-income countries. The findings of this real-patient study will provide insights based on the performance and usability of a prototype diagnostic decision support system in low- or middle-income countries.

**Trial Registration:**

ClinicalTrials.gov NCT04958577; http://clinicaltrials.gov/ct2/show/NCT04958577

**International Registered Report Identifier (IRRID):**

DERR1-10.2196/34298

## Introduction

### Background

Basic health care is insufficient for an estimated 4 billion people worldwide due to a global shortage of up to 7.2 million health care workers, which is expected to increase to a shortage of 12.9 million workers by 2035 [1]. The shortages in sub-Saharan Africa will be among the most profound because of a scarcity of medical schools in the region and an overall lack of training [1]. Additionally, poor clinical knowledge is a concern in regards to the level of care given worldwide [2]. A World Bank report found that 50% of rural health care workers were unable to diagnose 5 common conditions affecting patients in Tanzania [3]. Clinical deficiencies lead to a high number of excess deaths in low- and middle-income countries (LMICs) that would be prevented by the provision of higher-quality health care [4]. Clinical decision support systems (CDSSs), such as diagnostic decision support systems (DDSSs) can help to mitigate these problems. A few studies have investigated whether mobile phone–based CDSSs have enabled health care workers to provide better treatment, but thus far evidence is inconclusive [5]. It is therefore important to undertake further research on this topic in order to assess the potential role of mobile device–based CDSSs.

CDSSs are often implemented in real clinical settings and used by health care providers (HCPs) to aid decision-making at the point of care or for a specific care situation. The usefulness of CDSSs can be measured according to many different types of outcomes, such as clinical and health care processes or user workload and efficiency [6]. There is a great opportunity for CDSSs to improve patient outcomes with proper adherence; an example of these improved outcomes can be seen in one study, in which the final process composite score and patient satisfaction were higher in the patient group that used the CDSS compared to the group that did not [7]. One great challenge to CDSS and DDSS adoption and their clinical benefits is system usability. These systems must be practical and useful in a manner that supports their ongoing use by HCPs; this is a consistent challenge with almost all CDSSs [8,9].

### DDSS Evidence Base

A number of studies have explored the use of DDSSs in high-income countries (HIC) [10], where the rate of diagnostic error in clinical medicine is estimated to be as high as 15% to 50% [11]. A published systematic review of available DDSSs showed that they can provide accurate diagnostic suggestions [10], with a pooled accurate diagnosis retrieval rate of 70%. That review also presented preliminary evidence that small but significant improvements in physician diagnostic accuracy accompanied DDSS use. It has also been recognized, however, that many DDSSs provide long lists of suggested conditions, which might increase clinician uncertainty. Another barrier to DDSS uptake is the additional time required for their use. A systematic review by Riches et al [10] suggested that junior members of clinical teams, or those with less medical experience, input more data and were therefore more likely to benefit from the use of DDSSs. This is indicative of the potential of DDSSs in LMICs, where diagnoses are often made by HCPs with less formal medical training than medical doctors [3]. Overall, the review concluded that differential diagnosis generators have the potential to improve diagnostic practice among clinicians; however, the literature that they reviewed also revealed many caveats that must be considered in the application of these systems and their further development. For example, it was reported that accurate diagnosis retrieval alone does not predict the uptake or effectiveness of differential diagnosis generators in clinical settings, as there are other relevant characteristics that can influence uptake and effectiveness, including the specificity of the list of diagnoses, the time required to use the system, its availability and access, and its cost-effectiveness [10,12,13].

There have been fewer studies of DDSSs in LMIC settings. Although addressing diagnostic error is complex, suggested approaches and solutions include training in diagnostic techniques for clinicians and the use of electronic diagnostic aids, such as DDSSs, to augment the diagnostic abilities of doctors [10]. In a study conducted in Mexico that looked at a range of clinical patterns, a DDSS was shown to improve the diagnostic accuracy of family medicine residents for both rare and common illnesses alike; they achieved an accuracy of 82.4% (SD 8.5%) with the DDSS and 74.1% (SD 9.4%) without the DDSS [14].

DDSSs may have a role in upskilling health workers and in supporting doctors’ decision-making in the medium term. However, health-related artificial intelligence solutions developed in and for high-resource settings should not simply be used in an LMIC setting without adequate clinical investigation. Due to their development in settings with different health care structures and health care worker education levels, and their development on a base of medical data biased toward these settings, one cannot simply extrapolate data from HICs to LMICs. Instead, it is important to carry out clinical evaluations that specifically demonstrate the safety and performance of the technology for individual LMIC settings and its underlying medical reasoning before its use can be extended to new LMIC locations and use cases.

### Mid-level Health Care Workers

This study will be conducted in a district hospital in Tanzania, where the main entry point for patients into the health care system is primary care [15]. Primary care is delivered in an outpatient setting that includes dispensaries, health centers, and district hospitals [16]. In Tanzania, the health care staff shortage is severe; staffing is estimated to be 52% of the actual need [17]. Mid-level health care workers compensate for the insufficiency of qualified medical doctors by carrying out some aspects of the roles of doctors. Mid-level health care workers are a group predominantly found in LMICs [18]. They are defined as health care workers who undergo shorter training than physicians, but who nonetheless perform some roles generally considered part of a physician’s responsibilities [18]. In Tanzania, mid-level health care workers include clinical officers (COs) and assistant medical officers (AMOs) [19].

After graduating from secondary school, COs receive 3 years of practical and theoretical training. A CO’s responsibility is diagnosing and treating common conditions and performing minor surgeries. After gaining 3 years of practical experience, a CO can undergo a further 2 years of training to become an AMO. The position of an AMO involves a wider scope of medical practice; they can perform surgeries such as appendectomies and cesarean sections. Additionally, AMOs can act in leadership roles in medical facilities, especially in rural areas [19]. The prototype DDSS in our study will be used by COs. If there is no CO available for the patient consultation, then it will be used by an AMO. By evaluating the use of the prototype DDSS by mid-level HCPs, we aim to assess its potential to improve the performance of HCPs who have received less medical training than fully-qualified medical doctors.

### Objective and Hypotheses

This study is part 2 of the AFYA (Artificial Intelligence-Based Assessment of Health Symptoms in Tanzania) study series [20]. The current study will explore the potential of the prototype DDSS to empower HCPs in LMICs. The objective is to measure to what degree the prototype DDSS can enhance the diagnostic accuracy of mid-level HCPs. This will be done by comparing, in an observational study setting, the diagnostic accuracy of HCPs who use the study tool to HCPs performing usual care, henceforth referred to as usual HCP, and by comparing the accuracy of diagnoses submitted before and after input from the DDSS. Since impracticality is a common barrier to CDSS adoption [8,9], we will also collect qualitative data on the usability, usefulness, and acceptance of the prototype DDSS. These measurements will provide insights on the appropriateness of the prototype DDSS interface.

There are two study hypotheses: (1) a “chatbot”-based DDSS, which is similar to a symptom assessment app (SAA) as it asks a series of questions about the patient’s symptoms using a sequential “question flow,” is an appropriate interface for improving the accuracy of decision-making by mid-level HCPs in sub-Saharan Africa; and (2) the diagnostic accuracy of mid-level HCPs will be improved by the use of a “question flow” DDSS based on a chatbot.

## Methods

This study is the second of 2 studies in the AFYA study series, and is a prospective, 2-arm observational study conducted at Mbagala Rangi Tatu Hospital, Dar es Salaam, Tanzania. The first study was a general, prospective, observational assessment of a symptom assessment platform named “Ada” (Ada Health GmbH) that is used directly by patients. That study used the same clinical setting as the present study and a separate clinical study protocol that has been described previously [20].

The development of the trial protocol was conducted in accordance with the current Standard Protocol Items: Recommendations for Interventional Trials (SPIRIT) guidelines (Multimedia Appendix 1) [21].

### The Ada Prototype DDSS Specifications and Rationale of Use

The prototype DDSS evaluated in this study is being developed through an iterative user-centered design approach [22]. It has been modified from a CE (Conformité Européenne)-marked SAA [23] that was developed to provide laypeople the opportunity to determine what might be causing their health problems.

As part of the iterative user-centered process, we have explored different tools reported in various past studies, including by a study conducted in a British primary care waiting room that investigated the usability, acceptability, and utility of an SAA [24], a study conducted in a German emergency department that assessed urgent-care advice provided by an SAA [25], and another study in a German emergency department that gathered patients’ relevant symptoms and presented them to their physicians [26]. Additionally, Ada Health GmbH has developed a prototype HIC DDSS that uses the same underlying Ada medical intelligence platform but does not utilize a chatbot interface; it allows HCPs to update symptoms in real time without relying on a question flow. It also allows HCP-level clinical symptom and test-finding information to be entered. We have conducted careful formative usability research in collaboration with academic expert centers; this has also contributed to the iterative user-centered design process [27]. This HIC HCP-facing DDSS has shown potential for assisting early diagnosis of rare diseases [28], with the potential for economic benefits to health care systems [29] and potentially longer-term transformational benefits for rare disease management [30]. In cooperation with usability researchers and user interface designers, we have incorporated the findings from these studies into the further development of a DDSS prototype.

We determined that the HIC prototype DDSS explored in past studies [27-29] may not be optimal for low-resource settings, as it is less suited to the mobile phone interface and relies on physician training for effective use. We therefore explored the development of a simpler chat-based DDSS for lower-income settings, where the question-flow prompting of the HCP could provide clinical histories and diagnostic benefits.

**Figure 1:**
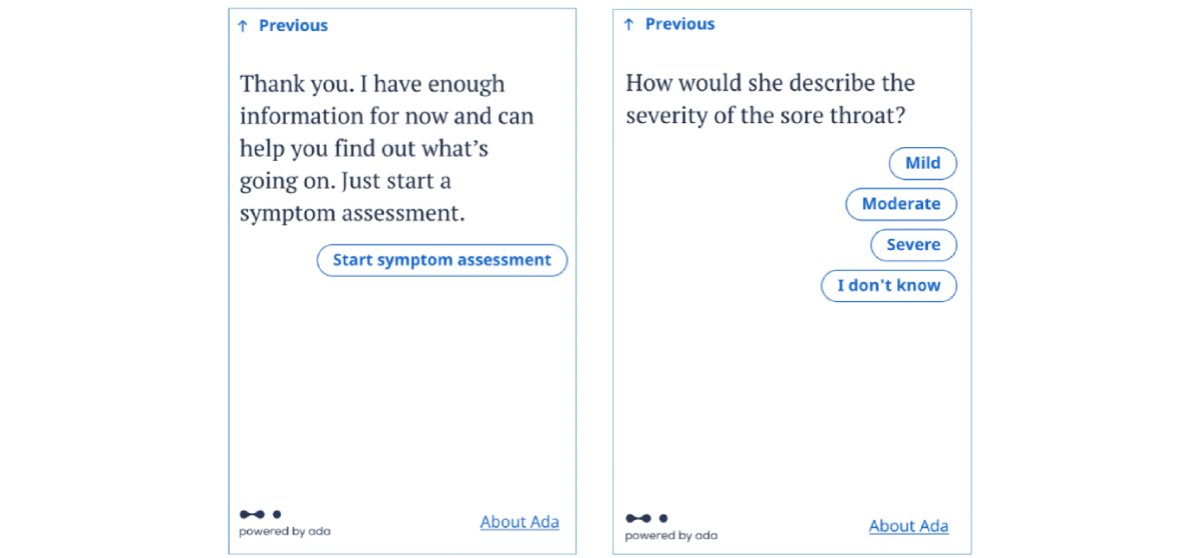
Screenshots showing the user interface of the tool in this study.

The prototype DDSS used in this study was created for use by LMIC health care workers, initially in an observational clinical study setting, to investigate the hypotheses of this study and to further understand the DDSS requirements of mid-level HCPs. The “chatbot” prototype DDSS interface asks the HCP to enter the patient’s age and their presenting complaint, followed by basic health information, such as the presence or absence of diabetes and hypertension, smoking, and pregnancy status (if relevant). It then asks a series of questions about the patient’s symptoms, with the optimal question asked at each point of the question flow so as to determine the most relevant information in a manner that is dynamically updated based on each answer provided by the patient. In this study, the chatbot is used on a handheld tablet, but it can be used in real world settings on any device with an internet connection, including smartphones, desktops, and laptops. The prototype DDSS will use the English language. This was decided after consultation with the HCPs involved in the study. The user interface of the study tool can be seen in Figure 1.

Dynamic questioning is delivered by the system’s underlying medical intelligence using a medical knowledge base built by medical doctors to define a Bayesian network. This Bayesian network allows approximate inference to be carried out. Based on a user’s answers, the system dynamically decides which questions to ask next in order to generate a list of potential conditions [24]. In this study, the Ada assessment was used to help mid-level HCPs gain relevant insights into the patient’s condition during their consultation by asking questions about the patient’s symptoms in an order determined by the system’s underlying reasoning engine. After the assessment, the HCP received a list of possible “condition suggestions” determined by the reasoning engine based on the patient’s presenting complaint and their symptoms. Along with these condition suggestions, the HCP was provided with additional information regarding the presentation of the disease, so that the HCP was able to make a more informed diagnosis.

The accuracy and comprehensiveness of the Ada platform has been validated in several studies of the Ada SAA [31-33]. In 2020, the Ada SAA, which uses the same medical reasoning as the prototype DDSS, was shown to be market leading in the accuracy of its condition suggestions and urgency advice compared to 7 other SAAs, and it had condition suggestions and safety performance comparable to United Kingdom general medical practitioners [31]. In a more recent study comparing 12 tools, the Ada SAA had the highest diagnostic accuracy at the first diagnosis (72%), while the next best SAAs achieved accuracies of 68% and 59.5%, respectively. Overall, the mean accuracy of all 12 SAAs was 37.7% [33]. When evaluating urgency advice, the Ada SAA was rated third best, with an accuracy of disposition of 64% (accuracy was 90% and 66.7%, respectively, for the first- and second-best SAAs), while the mean accuracy achieved by all SAAs was 57.7% [33].

### Study Optimization Phase

Before continuing on to the pilot study, there will be a feasibility and process optimization phase in which 15 patients will be recruited. This phase is for the optimization of general study procedures, patient tracking, and information recording in the busy clinical environment. In this phase, we will also be able to determine if staff training has been adequate and if the assessment is properly optimized for use by health care workers. Any deficiencies in the study process or staff training will be identified and a period of up to 2 weeks will be allowed for the rectification of any identified issues. Usual care for the patients will not change, and the data from these patients will not be included in the study analysis. After the optimization phase, at least 50 patients will be recruited for the pilot study.

### Patient Population and Eligibility Criteria

In this study, children, adolescents, and adults who arrive at the study site will be assessed for eligibility. The study site will be a busy district hospital waiting room at Mbagala Rangi Tatu Hospital in Dar es Salaam, Tanzania. Any person who enters the clinic and is willing or able to provide consent will be included, except for (1) patients who are not capable of completing a health assessment (eg, due to mental impairment, inebriation, or another incapacity) (2) patients with severe injury or illness that requires immediate treatment, or (3) patients with traumatic injury. Data from patients dropping out of the study or deviating from the protocol will be excluded from analysis.

In order to ensure that the study has a sample of patients with a comprehensive spectrum of symptom constellations and conditions, inclusion of patients will be monitored throughout the study; doing so will ensure that this pilot study does not just provide detailed testing for the most commonly presenting patient scenarios, but also provides testing for the performance of the prototype DDSS for a broad range of medical conditions. There will be a target of enrolling between 2 to 5 patients for conditions related to (1) abdominal pain or gastrointestinal issues, (2) the lower respiratory system, (3) the upper respiratory system, (4) mental health, (5) vision, (6) orthopedic issues, (7) the cardiovascular system, (8) the genitourinary system, (9) the neurological system, (10) the skin, (11) obstetrics and gynecology, and (12) the ear, nose, and throat. We plan to include at least one adult and one child in each category, where reasonable, and once a total of 5 patients have been enrolled for a given category, no further patients will be included. In cases in which the presenting complaint does not match the condition category with which the patient is ultimately diagnosed, the physician’s diagnosis will be aggregated on a dashboard adapted to optimize recruitment according to the categories listed above.

The maximum of 5 patients in one condition category is only an aim, but one that should be readily attainable. The study trackers hired for this study, that is, the workers who will recruit the patients, have CO and nurse midwife education and training, meaning that they are able, to a great extent, to determine these classifications.

### Interventions

This is an observational, prospective study. There will be no experimental or control interventions.

### Description of Study Visits and Assessment Schedule

**Figure 2:**
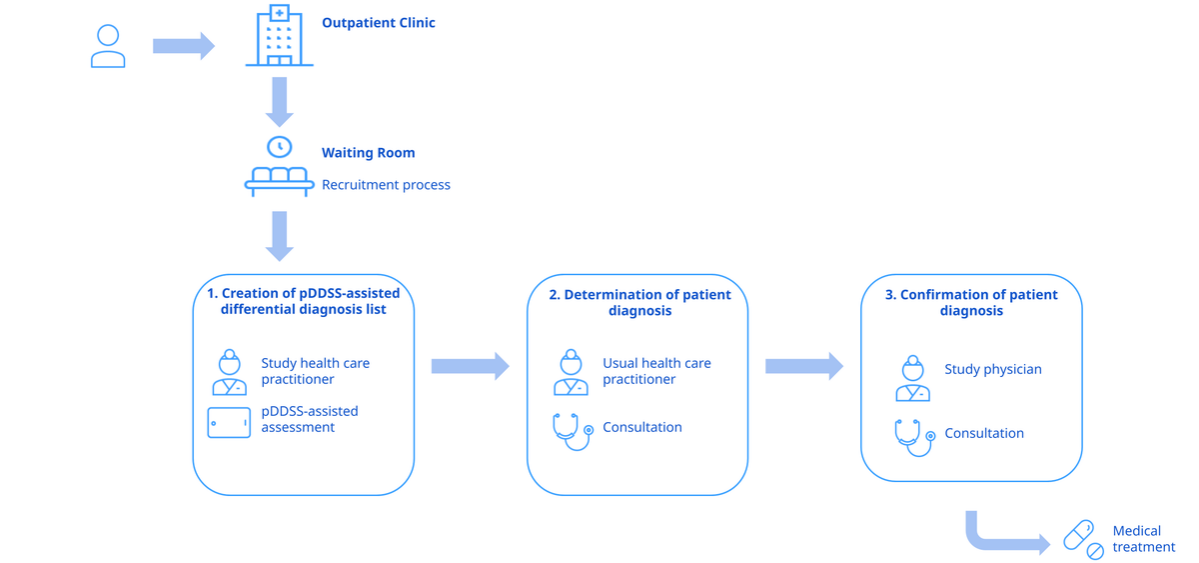
The patient journey in the study. Step 1: using the study tool, a differential diagnosis list is created by the study health care practitioner (clinical officer or assistant medical officer). Step 2: using a structured electronic case report form (eCRF), the patient consults with the usual health care practitioner for the determination of a diagnosis. Step 3: using a structured eCRF, the patient consults with a study physician to confirm the findings in step 2 with higher objectivity and a gold standard diagnosis. DDL: differential diagnosis list; pDDSS: prototype diagnostic decision support system.

The patient's journey in the study will consist of 3 stages (Figure 2 shows an overview).

### Patient Presenting to the Clinic (Recruitment Process)

For the recruitment process, the study staff will work in close cooperation with the hospital staff in order to identify potentially eligible patients in the waiting room. Patients assessed as potentially eligible will be approached by the study staff. The study staff will provide them with detailed information about the study and obtain written informed consent if the patients decide to enroll in the study. In addition to their parent’s or caretaker’s consent, children aged between 9 and 18 years will be requested to sign an assent form themselves. Enrolled patients will receive a unique study ID that is not part of the usual health care record and will be allocated alternately to the 2 study arms.

### Ada Assessment by Study HCP (Stage 1)

**Figure 3:**
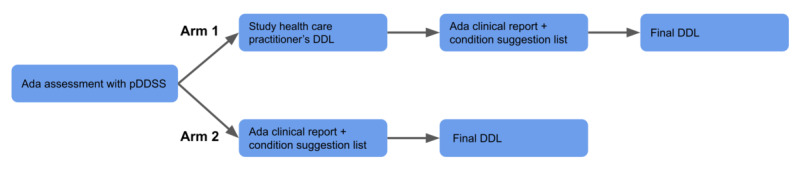
Comparison of study arms in stage 1. pDDSS: prototype diagnostic decision support system; DDL: differential diagnosis list.

After the recruitment process, the patients will consult with a study HCP, who is a mid-level HCP and will not be involved in the patient’s care. The study HCP will assess the patient using the prototype DDSS and create a differential diagnosis list. Depending on the patient allocation, there will be a difference in the synthesis of the final differential diagnosis list in the assessment (Figure 3): In arm 1, the study HCP will submit a preliminary list before seeing the prototype DDSS’s condition suggestion list. Once the preliminary list is submitted, the study HCP cannot change it. After being able to review the preliminary list and the prototype DDSS’s condition suggestion list, the study HCP will pick his or her top 5 differential diagnoses from both lists and submit a final differential diagnosis list. Conversely, in arm 2, the study HCP will see the prototype DDSS’s condition suggestion list before submitting a final differential diagnosis list. This will give the study HCP the opportunity to consider differential diagnoses from the prototype DDSS and add their own differential diagnoses to create a final differential diagnosis list. The degree to which these lists match the gold standard diagnoses will be judged in a later step by an unbiased physician panel, after the completion of patient recruitment and study data recording.

Our limit of 5 differential diagnoses is a pragmatic limit based on Ada’s limit of 5 condition suggestions; it is similar to the approach taken in many other papers in the medical literature, which have used comparisons with a gold standard diagnosis and a maximum of 5 to 10 differential diagnoses [33-35]. The reasoning for this is that the length of a diagnostic list has been found to be a key predictor of accurate diagnosis retrieval; long diagnostic lists are less specific and hence problematic for clinicians using differential diagnosis tools in a busy clinical setting [10].

### Patient Examination by Usual HCP (Stage 2)

Having finished step 1, the patients will continue on to a consultation with a usual HCP. In this step, the patient’s diagnosis and a further diagnostic plan for them will be determined as a part of usual care. The usual HCPs will be COs, AMOs, or medical doctors. The usual HCPs in stage 2 will not be the same HCPs that performed the interview in stage 1. During the consultation, the usual HCPs will fill out a standardized electronic form and a structured consultation report form through a tablet-based electronic case report form (eCRF). Afterwards, they will complete the standard hospital forms, record vital signs, and request laboratory or diagnostic procedures as part of usual care.

### Patient Examination by Study-Provided Physician (Stage 3)

After laboratory and diagnostic procedures, the patient will proceed to a consultation with a study-provided physician. This physician will, as in Stage 2, complete a structured consultation form through a tablet-based eCRF. The results of this consultation will amend the one from stage 2 in the gold standard panel. Adding a study physician not involved in the patient’s care to confirm the patient’s diagnosis ensures a higher degree of objectivity in the generation of the gold standard. The study physician will be able to refer to the usual HCP’s notes from stage 2, although this will be only allowed at the end of the consultation in order to minimize bias and ensure patient safety.

Although the interaction time between health care staff and the patient in the investigation will be about three times as long as the standard hospital process, this extra time will be compensated for by the study participants being moved forward in the waiting-room queue for examination, clinical testing, and receiving test results (when relevant). The overall effect is that the study participants will have a longer period of interaction at the clinic (with study trackers and study physicians), but will have a similar total visit length at the clinic (ie, less passive waiting time in the waiting room).

### Measurement Methods

**Figure 4:**
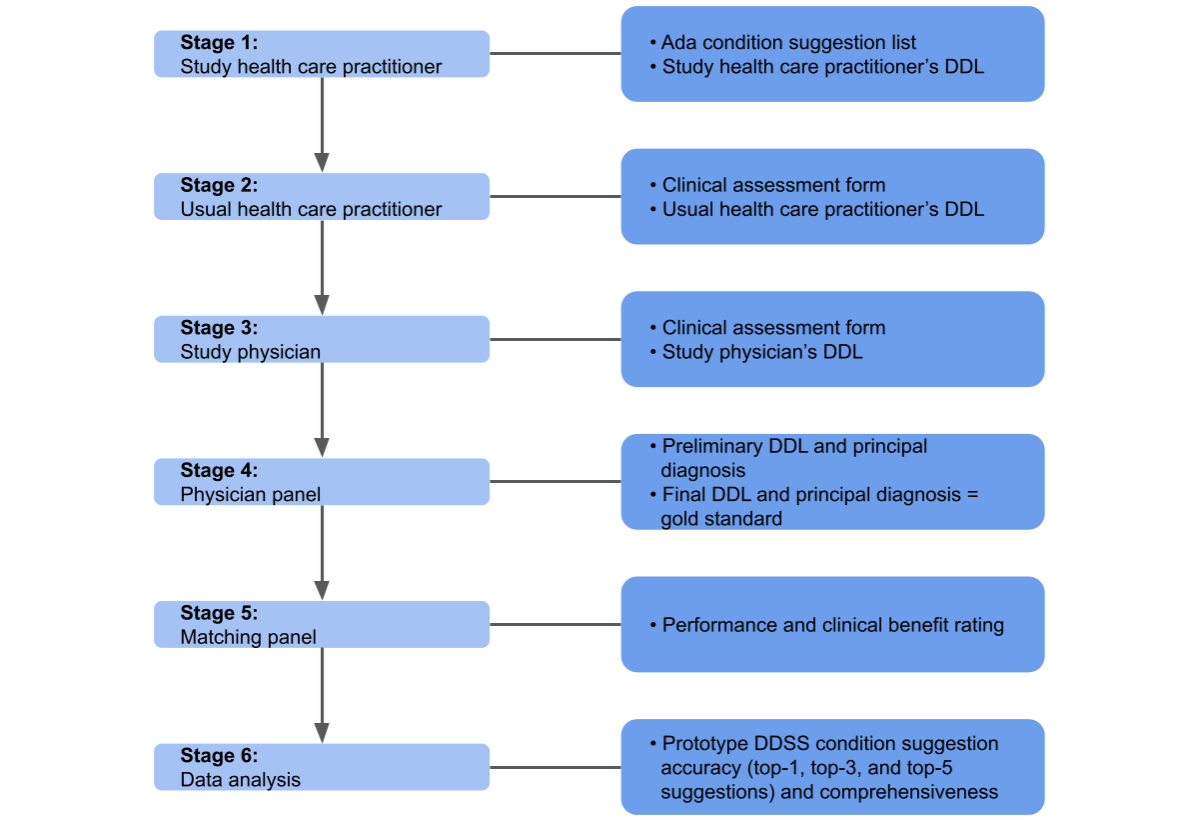
Data flowchart. DDL: differential diagnosis list; DDSS: diagnostic decision support system.

Overall, there will be 5 stages in the process of data collection and physician panel assessment (Figure 4 shows an overview). The first 3 stages in this process have already been described: (1) the study HCP will use the prototype DDSS; (2) the patient will consult with a usual HCP; and (3) the patient will consult with the study-provided physician. There are then 2 additional steps: (4) generation of a differential diagnosis by the physician panel, and (5) matching of conditions by the physician panel.

The physician panel in step 4 that makes the differential diagnoses will be made up of 3 local physicians, each with at least 3 years of full-time clinical experience (the majority in general medicine), who will carry out a review process; this process will yield the gold standard differential diagnosis list for each case. The physician panel members have been thoroughly screened and chosen by the principal investigator of this study, who is a native Tanzanian doctor with over 20 years of experience as a medical doctor and several additional years of experience as an instructor at Muhimbili University of Health and Allied Sciences. All physician panelists are required to have an active medical license and to be available for at least 9 hours per week during the physician panel process in order to be immersed enough in the process. Two independent “reviewer” physicians on the panel will review the diagnoses from the usual HCP and the study-provided physician, the pseudo-anonymized medical history, and the symptoms of each patient. They will assign a preliminary differential diagnosis list and a principal diagnosis to each case based on history and symptoms alone and then assign a final differential diagnosis list and principal diagnosis based on the full patient clinical file (this will include vital signs and results from medical examinations and diagnostic tests). In case of disagreement between the 2 differential diagnosis lists, a third reviewer will make a final decision. The tenth revision of the International Statistical Classification of Diseases and Related Health Problems will be used to determine all diagnoses.

The matching of conditions by the physician panel in step 5 will involve matching the diagnoses made by the usual HCP and study-provided physician with the diagnoses made by the reviewer physicians on the physician panel. The same procedure for the DDSS condition list will be carried out by the reviewer physicians. As in step 4, a third reviewer physician will make a final decision if there is disagreement between the 2 reviewer physicians. The primary comparator of the Ada assessment is the differential diagnosis obtained before the addition of vital sign measurement, physical examination, and additional diagnostic tests. Looking at the differential diagnosis list at each point in the patient’s clinical journey is still a relevant point of comparison.

Finally, the data collection performed in these 5 steps will be analyzed (details are in the “Data Analysis” section). In order to determine the usability, usefulness, and acceptability of the prototype DDSS, the usual HCP questionnaires, in addition to the patient questionnaires, will be analyzed and described using methods appropriate to modified Likert-scale questionnaire data [26].

### Endpoints

#### Primary Endpoints

The first endpoint will be the condition suggestion accuracy and comprehensiveness of the prototype DDSS, evaluated against the gold standard differential diagnosis determined by the review panel, reported in the context of the accuracy of the usual HCP. The second endpoint will be the study HCP’s condition suggestion accuracy and comprehensiveness, which will be compared between arm 1 (in which the HCPs determine their own list of condition suggestions before seeing the prototype DDSS’s condition suggestions, then create a final ranked list of condition suggestions) and arm 2 (in which they see the prototype DDSS’s condition suggestions before determining their list of overall ranked condition suggestions). The third endpoint will be a comparison of the study HCP's condition suggestion accuracy and comprehensiveness in arm 1 in the preliminary and final DDLs.

#### Additional Data of Interest

Additional data of interest will include qualitative data on the usability, usefulness, and acceptability of the prototype DDSS.

### Outcomes and Outcome Measures

The design of this study is based on the literature on pilot study design, as it will serve as a guide for a larger trial in the future [36,37]. The sample size of a minimum of 50 participants was a pragmatic determination of a number of participants that could be used to assess the ability to recruit patients across a spectrum of conditions in a clinic of this type and in this setting, to evaluate the feasibility of collection of the complete study data, and to allow an accurate analysis. There are multiple aspects of study design and operation that will be explored in this pilot study: (1) investigating if it is feasible to determine the accuracy and comprehensiveness of the Ada DDSS for a large range of symptoms and conditions in multiple different age groups; (2) establishing how many patients and HCPs can be recruited and the likely number of completed patient and HCP questionnaires; (3) trialing new methods and enabling power calculations intended to be used in a later single- or multicenter randomized controlled trial; and (4) evaluating the general feasibility, both technical and logistical, of a full-scale study, including issues of data collection and questionnaire design. The pilot has not been powered to definitively accept or reject the scientific hypotheses, as a pilot is required before the sample size for a definitive study can be estimated.

### Risk-Benefit Assessment

This study does not pose any risk to the patients, as it is solely observational; therefore, there is no need for additional safety management. Patients requiring immediate medical care and clinically unstable patients will be excluded. If patients are called into their appointment before the study HCP finishes the prototype DDSS assessment, they will be excluded from the study process and analysis and proceed to usual care, meaning there will be no delay in the diagnosis or treatment of any patient. Although there will be 2 extra consultations for patients enrolled in the study, these consultations will generally require less than 10 minutes; therefore, it is highly unlikely to delay a patient’s diagnosis or treatment.

### Data Management and Data Safety

This study has a data management plan that outlines the guidelines by which data entry will take place. All consent forms will be paper based; the rest of the data will be collected electronically. Paper records will be kept in a locked cabinet in the facility only accessible by study-specific personnel, while for electronic data, a clinical-trial electronic data capture (EDC) system (REDCap) will be used. Overall, study-site data collection will go through a secured local area network, allowing data sharing on site. Study personnel, who will be provided unique usernames and passwords, will be trained on the EDC system. Before committing data to the EDC system, the research assistant will first verify that the data are correct. After this, the research assistant will not have access to the data, as they will be automatically locked after each commit. Storage of the data will last a minimum of 3 years from the date that the last patient is seen at Muhimbili University of Health and Allied Sciences; 10% of the digitized usual care consultation notes will undergo source-data verification.

### Data Analysis

Applying the definition from Gilbert et al [31], we will assess the accuracy and comprehensiveness of the top-1, top-3, and top-5 suggested conditions by the prototype DDSS in comparison to the gold standard differential diagnoses. Data analysis will also be carried out as presented by Gilbert. In short, descriptive tests and statistics appropriate for categorical data will be utilized to compare condition suggestion accuracy. To analyze if the proportion of correct condition suggestions from the prototype DDSS, from the usual HCPs, and from the study-provided physicians are drawn from the same distributions, the chi-squared test will be applied. If there is a significant difference, we will apply a 2-sided post hoc pairwise Fisher exact test to compare the prototype DDSS and the practitioners. Due to the low sample size of this pilot study, it may be the case that the chi-squared tests will not produce significant results. As the study goal is the comparison of the prototype DDSS to usual care, the ratings of the usual HCPs will be strictly anonymized.

### Patient and Public Involvement

No patients or members of the public were directly involved in the development of the research hypotheses or study design. However, feedback and learnings from patients and other individuals from past, related studies of the Ada SAA, as well as previous studies carried out at the study site, were used to help design the study and patient interaction protocols [24,26,27,31].

### Ethics Approval

The pilot study received ethics approval from the ethics committee of Muhimbili University of Health and Allied Sciences (MUHAS-REC-09-2019-044) and the National Institute for Medical Research (NIMR/HQ/R.8c/Vol. I/922). All amendments to the protocol have been reported and adapted on the basis of the requirements of the ethics committee. The trial has been registered at ClinicalTrials.gov with the registration number NCT04958577.

## Results

Patient recruitment started in October 2021 in the outpatient clinic of the Mbagala Rangi Tatu Hospital. Participants will be recruited directly in the waiting room of the outpatient clinic. Data collection will conclude in May 2022. Data analysis is planned to be finished by the end of June 2022. The results will be submitted to peer-reviewed journals and local and international stakeholders and will be communicated in editorials and articles by Ada Health GmbH.

## Discussion

As this is the first study of a chatbot-based DDSS on a broad range of conditions conducted in an LMIC, we expect that our pilot results will show variable accuracy compared to DDSSs used in HICs. Nevertheless, we expect good overall usability and acceptance of the prototype.

### Development of a New DDSS for LMICs

While health care systems in HICs can also benefit from DDSSs, there is a substantially higher need for improvement in the delivery of qualitative health care in LMICs [2,4]. Due to their high scalability, digital solutions such as mobile health tools can be cost-effective in mitigating these problems. Web-based applications have hardware requirements that are quite low. Internet connectivity is still required, but while there are gaps, especially in rural settings, the quality of internet connectivity is increasing in many LMIC settings [38]. Additionally, this approach makes it possible to update the DDSS without extensive maintenance on the HCP side. Artificial intelligence–based and short message service–based health tools have already been used in different areas of medicine in Africa [39-42].

Widespread adoption of CDSSs requires high usability and integration into the clinical workflow with minimal disruption. This study is an important step in the iterative, user-centered design process that we will use to develop a DDSS for LMIC health care workers. On one hand, this study will provide insights into the accuracy of the app and enable us to improve our medical database based on real-world experience in the setting of an African clinic. On the other hand, the qualitative data on the usability, usefulness, and acceptability of the prototype DDSS will give us the opportunity to further develop the current prototype to match the needs of our target users (health care workers, in this case) and thus increase acceptance of the tool.

This study will test one cycle of a design process and will gather further information on the appropriateness of a chatbot-based DDSS for mid-level HCPs. The study outcomes will allow further ideation and definition of the requirements in later prototypes.

### Strengths and Limitations of the Current Study

There are several strengths and limitations to this study. It is prospective, unlike previous assessments of DDSS performance, which have mostly been retrospective [10]. The Tanzanian outpatient clinic setting will provide a heterogeneous patient population. Through the application of broad inclusion criteria, we aim to include a diverse patient population with varied conditions covering a wide range of age groups. Another strength of this design is the approach to determining the gold standard diagnosis, which is based on the methods in Gilbert et al [31] and Semigran et al [43] and will use a physician panel and voting methods to achieve high objectivity and independence.

This is a single-site study in the outpatient clinic of a Tanzanian district hospital; therefore, the study’s findings may not be applicable to other LMICs. The relatively small enrollment target of a minimum of 50 participants is another limitation, but as this is a pilot study, this sample size is adequate to enable the design of a definitive later study. The study questionnaires filled out by the participants and the usual HCPs are not based on previously validated instruments and thus response bias cannot be excluded.

Considering the pilot character of this study, the limitations are appropriate, as the findings of this study can be used in the conception of a larger trial that will validate the accuracy and usefulness of a chatbot-based DDSS.

## Appendix

Multimedia Appendix 1 SPIRIT checklist.

## Abbreviations

AFYA: Artificial Intelligence-Based Assessment of Health Symptoms in Tanzania
AMO: assistant medical officer
CDSS: clinical decision support system
CO: clinical officer
DDSS: diagnostic decision support system
eCRF: electronic case report form
EDC: electronic data capture
HCP: health care practitioner
HIC: high-income country
LMIC: low- and middle-income countries
SAA: symptom assessment app
